# Effects of Different Light Wavelengths on Fruit Quality and Gene Expression of Anthocyanin Biosynthesis in Blueberry (*Vaccinium corymbosm*)

**DOI:** 10.3390/cells12091225

**Published:** 2023-04-23

**Authors:** Zhiwen Wei, Haiyan Yang, Jie Shi, Yongkang Duan, Wenlong Wu, Lianfei Lyu, Weilin Li

**Affiliations:** 1 Co-Innovation Center for Sustainable Forestry in Southern China, College of Forestry, Nanjing Forestry University, Nanjing 210037, China; 2 Institute of Botany, Jiangsu Province and Chinese Academy of Sciences (Nanjing Botanical Garden Mem. Sun Yat-Sen), Jiangsu Key Laboratory for the Research and Utilization of Plant Resources, Nanjing 210014, China

**Keywords:** light wavelength, blueberry, fruit quality, anthocyanin, antioxidant capacity

## Abstract

Different light wavelengths display diverse effects on fruit quality formation and anthocyanin biosynthesis. Blueberry is a kind of fruit rich in anthocyanin with important economic and nutritional values. This study explored the effects of different light wavelengths (white (W), red (R), blue (B) and yellow (Y)) on fruit quality and gene expression of anthocyanin biosynthesis in blueberry. We found that the B and W treatments attained the maximum values of fruit width, fruit height and fruit weight in blueberry fruits. The R treatment attained the maximum activities of superoxide dismutase (SOD) and peroxidase (POD), and the Y treatment displayed the maximum contents of ascorbic acid (AsA), glutathione (GSH) and total phenol in fruits, thus improving blueberry-fruit antioxidant capacity. Interestingly, there were differences in the solidity–acid ratio of fruit under different light-wavelength treatments. Moreover, blue light could significantly improve the expression levels of anthocyanin biosynthesis genes and anthocyanin content in fruits. Correlation and principal component analysis showed that total acid content and antioxidant enzymes were significantly negatively correlated with anthocyanin content in blueberry fruits. These results provide new insights for the application of light wavelength to improve blueberry fruit quality and anthocyanin content.

## 1. Introduction

Blueberry (*Vaccinium* spp.) is a small, perennial berry fruit tree, which is native to North America. As an important cash crop, the planting area of blueberry is increasing all over the world, and its fruit has a unique flavor and high nutritional value. Blueberry fruits not only contain carbohydrates, amino acids, vitamins and other nutrients, but they are also rich in bioactive substances such as polyphenols and anthocyanins [1]. Interestingly, blueberry fruits are rich in anthocyanins, which give them a dark blue color when ripe. Polyphenols and anthocyanins are powerful antioxidants, which could strengthen antioxidant capacity and lower oxidative stress in plants [2]. Consumers have an obvious preference for fruits with high anthocyanins and polyphenol contents, which are beneficial for the prevention of multiple cancers, cardiovascular protection and for their antioxidant and other health benefits [3]. Thus, with the increasing attention to human health and the crucial function of anthocyanins in plants, people are more and more interested in anthocyanins. Anthocyanins are common pigments in fruits and flowers and a class of flavonoid compounds, which are biosynthesized through a phenylpropanoid pathway. Anthocyanins generally accumulate rapidly during fruit ripening, with the highest level in ripe fruits, and their stability is affected by light, temperature, PH and other factors [4,5]. The major anthocyanins in blueberry include cyanidin, peonidin, petunidin, delphinidin, malvidin and pelargonidin [6]. In plants, the biosynthetic pathways of anthocyanins have been fully studied, and some structural genes related to the pathway in blueberry have been identified [7]. Moreover, the key enzymes involved in anthocyanin biosynthesis encoded by these genes mainly include the following: cinnamate 4-hydroxylase (C4H), 4-coumarate-CoA ligase (4CL), chalcone isomerase (CHI), dihydroflavonol-4-reductase (DFR), UDP-glucose flavonoid glucosyltransferase (UFGT) and leucoanthocyanidin dioxygenase (LDOX) [8,9,10]. The upregulated expression of these structural genes could improve the activities of anthocyanin-biosynthesis enzymes, thus increasing the level of anthocyanins in plants. Different genotypes and developmental stages are important factors to determine the type and level of anthocyanins in blueberry fruits. In addition, cultivation measures also play key roles in improving anthocyanin content and fruit quality of blueberry.

At present, even though the fruit quality and anthocyanin content of blueberry can overall meet the demands of production and consumers, they still need to be improved continuously. Previous studies on the impact of blueberry fruit quality focused on breeding improvement, fertilizer application and substrate cultivation, which further developed the blueberry industry [11]. However, environmental factors also have an important impact on fruit quality, especially the level of anthocyanins in fruits [2,8,12]. Light is an essential environmental factor for plants in nature, which not only affects plant growth and development, but also plays a key role in the formation of fruit quality [8,9,13]. In order to adapt light intensity, light wavelength, photoperiod and other light characteristics, plants have evolved different photoreceptors to sense a variety of light signals [14,15]. Generally, wavelengths in the range of 280 to 750 nm could be perceived and further transduced to special signal using visible photoreceptors and UV-B photoreceptors [16]. Cryptochromes (CRYs) and phototropins (PHOTs) are blue-light photoreceptors, and red light is absorbed by phytochromes (PHYs) [14]. As an energy source for carbohydrate production in photosynthesis, light can also regulate many physiological and metabolic activities of plants through different signal pathways, such as secondary metabolism, antioxidant defense system and photosynthesis [17,18,19]. Moreover, fruit coloration is also affected by light, and light regulates the biosynthesis of anthocyanins, carotenoids and other color-producing substances [20,21]. It has been demonstrated that light could directly affect the accumulation of anthocyanins in plants, but this largely depends on light intensity and light wavelength [15]. It is generally believed that increasing the intensity of light can lead to better coloring and accumulation of anthocyanins in fruits [22], but it is also related to light wavelength. In all visible light spectra, blue light and red light are considered to have the most significant impact on plant growth and fruit development [23]. Additionally, white light and yellow light also affect fruit quality. In recent years, many researchers have reported that light wavelength has an obvious impact on anthocyanins level in fruits. For example, Lekkham et al. found that red light could improve anthocyanin levels in apple by inducing the biosynthesis of anthocyanins [24]. Similarly, a study on grape also revealed that blue light induced the expression of anthocyanin-biosynthesis genes, thereby increasing anthocyanin content in fruits [13]. Therefore, different light wavelengths show different regulatory mechanisms for anthocyanin biosynthesis in plants.

Light emitting diodes (LEDs) have the advantages of being energy-saving, lightweight, durable and they produce less heat, are highly efficient, consume low amounts of energy in comparison to traditional light sources and are more widely used in greenhouses for the commercial production of horticultural crops [25]. Previous studies have focused on the effect of light wavelength on the quality of traditional crops, such as apples, tomatoes and grapes, but there are few studies on the impact of different light wavelengths on the fruit quality and anthocyanin level of blueberries. In particular, the regulatory mechanism of light wavelength on anthocyanin biosynthesis in blueberry fruit is still unclear. In this study, we utilized LEDs to provide different light sources to explore the effects of different light wavelengths on appearance, flavor, antioxidant capacity, bioactive substance and anthocyanin biosynthesis gene expression level in blueberry fruit. Therefore, the results of this study could supply new insights into the regulatory mechanism of light wavelength on anthocyanin biosynthesis in blueberry fruits and have certain reference value for the application of light wavelengths in greenhouses to improve the fruit quality of small berries.

## 2. Materials and Methods

### 2.1. Plant Materials and Light Treatments

This experiment was carried out in a greenhouse located at the Institute of Botany, Jiangsu Province, and the Chinese Academy of Sciences (E118°41′, N32°36′) from 10 April 2020 to the end of June 2020. Two-year-old healthy plants with uniform size of the southern highbush blueberry cultivar ‘Star’ (*Vaccinium corymbosum*) were selected as research materials and potted in an organic substrate (coconut coir:peat:perlite = 4:4:2). ‘Star’ originated from the cross FL80-31 × O’Neal. The growth conditions were as follows: average temperature, 26.7 °C; relative humidity, 65–75%; photoperiod, 12-h light and 12-h dark and light intensity, 300 µmol·m^−2^·s^−1^.

The blueberry plants were treated with four different light wavelength treatments in this experiment: full red light (R, 660 nm), full blue light (B, 460 nm), full yellow light (Y, 590 nm) and white light (W) used as the control. The light sources of all treatments were supplied by LED light (length, 1.2 m; power, 20–40 W and wavelength, 380–800 nm). The LED light sources were placed on the top of a culture rack with a steel frame structure, and silver shading cloth was laid on the outer culture rack to isolate different treatments. The light intensity could be adjusted by changing the distance between the plant and the light source, and light intensity was measured using a 3415FX photometer (Spectrum Technologies company, Dallas, TX, USA). Each treatment contained three biological replicates, and other cultivation and management procedures were consistent except for the use of different light wavelengths. The blueberry plants were fertilized every two days and watered once a week. The fertilizer was multi-nutrition and water soluble and produced from Shandong Jining Jinshan Biological Engineering Co., Ltd. in China. The nutrients of the fertilizer were as follows: 20% N, 20% P, 20% K, 0.21% B, 0.03% Zn, 0.04% Ca, 0.10% Fe, 0.01% Mn, 0.005% Cu and 0.002% Mo. Before application, we diluted the fertilizer one thousand times and adjusted its PH value to 5.0 with citric acid. We installed insect-proof nets, stuck yellow sticky traps in the greenhouse and sprayed pesticides on plants to prevent pests and diseases. We sprayed the blueberry plants with a mixture of carbendazim and dichlorvos once a month and applied an appropriate of amount of Beauveria bassiana powder to the base of plants. Fruit samples from each treatment were collected after 0, 15, 30, 45 and 60 days from the beginning of the light treatments and stored at −80 °C until further biochemical analysis.

### 2.2. Measurement of Fruit Appearance Indexes and Firmness, Soluble Solids and Total Acid

The fruit width and fruit height were measured using a Vernier calliper. The fruit weight was measured using an electronic balance. The ratio of the fruit height to the fruit width of blueberry fruit was represented by the shape index. The firmness of fruits at 60 d was measured using a fruit firmness tester (Catalogue No. 9300 (KM-5); Takemura Electric Works, Ltd., Kyoto, Japan). The probe diameter was 1 mm, and the pressing distance was 5 mm. The firmness was represented by the maximum breaking force in units of kg/cm^2^. The soluble solids content was measured using a handheld refractometer (Atago, WYT-A, Tokyo, Japan). The total acid content in the blueberry fruits was calculated as citric acid, according to the determination of total acid in foods (GB/T12456-2008). In addition, the solidity–acid ratio was used to represent the ratio of soluble solids content to total acid content.

### 2.3. Determination of Total Phenol and Total Anthocyanin Content

Fruits were collected at 60 d in all treatments to determine total phenol and anthocyanin contents. The anthocyanin content was determined using a modified pH difference method [26]. The total phenol content was determined using the Folin–Ciocalteu method with slight modifications [27].

### 2.4. Determination of Antioxidant System-Related Indexes 

The determination method for superoxide anion radical (O_2_^·−^) production rate was described by Wang et al. [28]. The malondialdehyde (MDA) content of blueberry fruit was determined according to the thiobarbituric acid (TBA) method [29]. The superoxide dismutase (SOD) activity in the fruits was determined according to the method of Stewart and Bewley [30]. The contents of hydrogen peroxide (H_2_O_2_), ascorbic acid (AsA) and glutathione (GSH), as well as the activity of peroxidase (POD), were determined using test kit protocols (Nanjing Jiancheng Institute of Bioengineering, Nanjing, China).

### 2.5. Gene Expression Analysis

Fruit samples at 60 d in each treatment were collected for gene expression analysis. Total RNA was extracted using a total RNA extraction kit (Bioteke, Beijing, China). The cDNA was synthesized with a cDNA synthesis kit (Pudi, Shanghai, China). The gene expression levels were analyzed with a qTOWER 2.2 real-time quantitative PCR system (Analytik, Jena, Germany) using a SYBR Premix Ex Taq^TM^ kit (Takara, Dalian, China). Each qRT-PCR reaction mixture comprised 1 µL of cDNA, 10 µL of TB Green Premix Ex Taq, 0.5 µL of each primer and 8 µL of RNase-free water in a total volume of 20 µL. The parameters of the amplification program were set as follows: 95 °C for 2 min, 95 °C for 10 s, 60 °C for 10 s, 72 °C for 15 s and melt for 6 s for a total of 40 cycles. Three replicates were performed using qRT-PCR in each treatment. The relative expression levels of related genes were calculated using the 2^-ΔΔCT^ method. The primer sequences for qRT-PCR were designed with Primer 6.0 (Table 1).

### 2.6. Statistical Analysis

One-way analysis of variance was used to analyze the data with IBM SPSS Statistics v.25.0, and Duncan’s multiple range test was used to determine significant differences among the treatments, and significance was set to *p* < 0.05. The correlation analysis and principal component analysis (PCA) were conducted on Origin 2022 (Origin Lab, Inc., Northampton, MA, USA).

## 3. Results

### 3.1. Fruit Appearance Indexes

As shown in Table 2, the appearance of blueberry fruits responded to different light wavelengths; the values of fruit width, fruit height and fruit weight of blueberry fruits increased with fruit ripening and significant changes were seen in different light wavelength treatments at different developmental stages. At 60 d, the three indexes treated with W light and B light reached their maximum levels in the fruits, and they were significantly higher compared to the other treatments, whereas the minimum values of these indexes were found in the R treatment. The fruit shape index could be used to assess the fruit shape. There were significant differences in the fruit shape index among the different treatments at 15, 3, and 45 d, while no significant changes in this index were observed under different light wavelengths at 60 d.

### 3.2. Generation Rate of O_2_^·−^, H_2_O_2_ and MDA Contents

**Figure 1:**
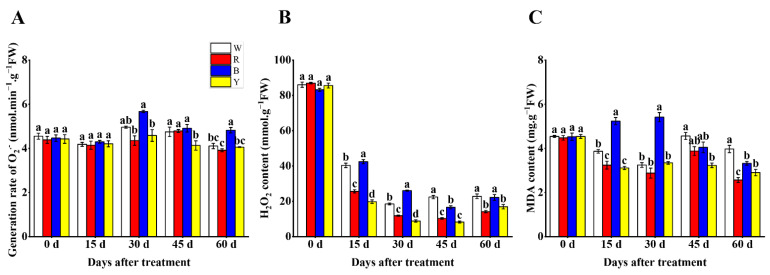
Effects of different light wavelengths on generation rate of O_2_^·−^ (**A**), H_2_O_2_ content (**B**) and MDA content (**C**) in blueberry fruit at 0, 15, 30, 45 and 60 d. All values are expressed as the means ± SDs. Different letters indicate significant differences among the treatments at *p* < 0.05.

The accumulation of O_2_^·−^, H_2_O_2_ and MDA in plants is usually used to assess the extent of cell damage. The maximum generation rate of O_2_^·−^ was observed in the B treatment and was significantly higher compared to other treatments during blueberry fruit ripening, whereas those of the R and Y treatments were relatively lower (Figure 1A). With blueberry fruit ripening, the H_2_O_2_ content showed an overall decreasing trend under different treatments, and significant decreases in H_2_O_2_ content were seen in fruits treated with Y light and R light, while the fruits treated with W light and B light showed relatively higher H_2_O_2_ contents at different developmental stages (Figure 1B). The Y and R treatments exhibited significantly lower MDA content in fruits during the whole fruit development period, whereas significant increases in MDA content were found in the B treatment at 15 and 30 d, and the W treatment displayed the maximum MDA content at 45 and 60 d in comparison to other treatments (Figure 1C).

### 3.3. Antioxidant Capacity

**Figure 2:**
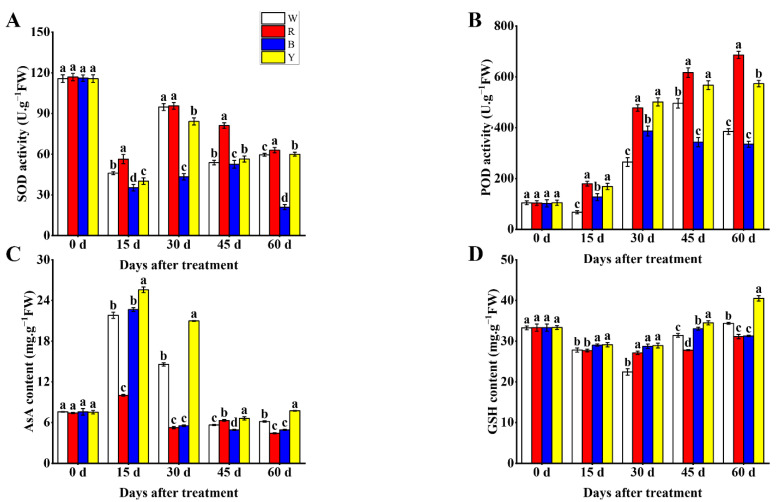
Effects of different light wavelengths on SOD activity (**A**), POD activity (**B**), AsA content (**C**) and GSH content (**D**) in blueberry fruit at 0, 15, 30, 45 and 60 d. All values are expressed as the means ± SDs. Different letters indicate significant differences among the treatments at *p* < 0.05.

SOD and POD are key antioxidant enzymes in the plant antioxidant system. There were significant changes in SOD activity when fruits were treated with different light wavelengths at different developmental periods. The SOD activity of fruits treated with R light was significantly higher in comparison to other treatments at 15, 45 and 60 d, and the B treatment showed the lowest SOD activity during the whole fruit development process (Figure 2A). The POD activity of fruits changed significantly under different treatments throughout fruit development. Significantly higher POD activities in the R and Y treatments were obtained in fruits relative to other treatments at different developmental periods, and the maximum activity of POD was seen in the R treatment, followed by the Y treatment at 45 and 60 d, whereas the B treatment maintained relatively lower POD activity with fruit ripening (Figure 2B). AsA and GSH are also antioxidants that exhibit significant effects in reducing oxidative stress. Significant differences in AsA content of fruits were observed under different light wavelength treatments at 15, 30, 45 and 60 d, but the difference of AsA content among different treatments gradually decreased with fruit ripening, and the Y treatment maintained significantly higher AsA content in blueberry fruits compared to other treatments, and significant increases were observed in the R and B treatments during the whole fruit development period (Figure 2C). Similarly, the overall change range of GSH content among different treatments was significant at different developmental stages, and significantly higher content of fruits treated with Y light were seen compared to other treatments; however, the R treatment displayed relatively lower GSH content in fruits at 45 and 60 d (Figure 2D).

### 3.4. Flavor Indexes and Bioactive Substances

**Figure 3:**
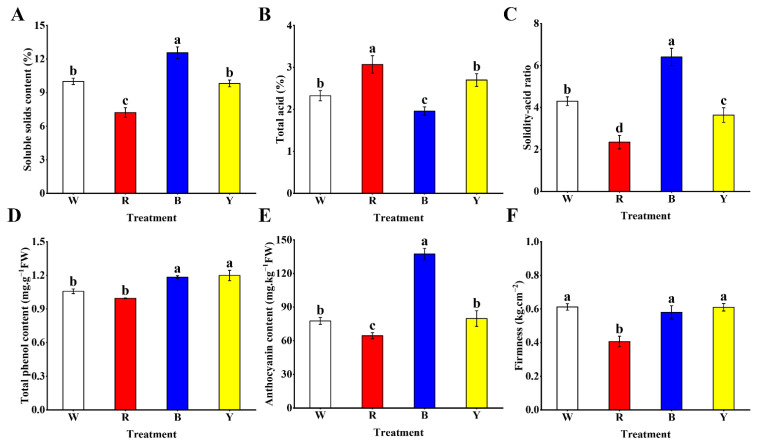
Effects of different light wavelengths on soluble solids content (**A**), total acid (**B**), solidity–acid ratio (**C**), total phenol content (**D**), anthocyanin content (**E**) and firmness (**F**) in blueberry fruit at 60 d. All values are expressed as the means ± SDs. Different letters indicate significant differences among the treatments at *p* < 0.05.

To investigate the impact of different light wavelengths on the flavor of blueberry fruit, the contents of soluble solids and total acid in each treatment were analyzed. There was no significant change in soluble solids content between the W and Y treatments, but these were significantly lower than that of the B treatment, and the fruits treated with R light exhibited a significant decrease in soluble solids content compared with other light wavelengths (Figure 3A). Likewise, the total acid content of blueberry fruits varied with the light wavelength and displayed the following trend: R > Y > W > B (Figure 3B). Generally, the solid–acid ratio also plays a vital role in fruit flavor. The maximum value of the solid–acid ratio was seen in the B treatment, whereas the R treatment attained the lowest value of the solid–acid ratio (Figure 3C). Polyphenols and anthocyanins are important nutrients and bioactive substances in blueberry fruits. The total phenol content of fruits in the Y and B treatments displayed no significant change, but were significantly higher compared to other treatments, and the minimum of total phenol content was obtained in fruits treated with R light (Figure 3D). Furthermore, the B treatment attained the maximum anthocyanin content in fruits, followed by the Y and B treatments, whereas that of the R treatment was significantly lower relative to other treatments, particularly the value for the B treatment, which was at least 70% higher than other treatments (Figure 3E). Interestingly, the minimum value of firmness was seen in fruits treated with R light and showed a significant decrease in comparison to other treatments, but no significant changes in firmness among the other treatments were seen (Figure 3F).

### 3.5. Expression Analysis of Genes Related to the Anthocyanins Biosynthesis Pathway

**Figure 4:**
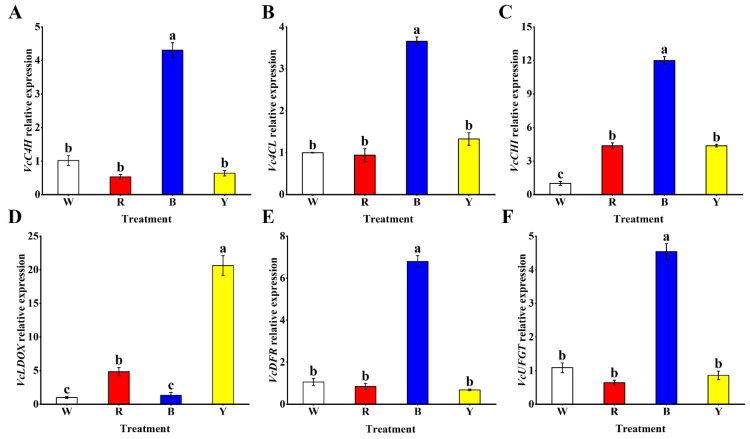
Effects of different light wavelengths on expression levels of genes related to anthocyanin biosynthesis: (**A**) *VcC4H*, (**B**) *Vc4CL*, (**C**) *VcCHI*, (**D**) *VcLDOX*, (**E**) *VcDFR* and (**F**) *VcUFGT* in blueberry fruit at 60 d. All values are expressed as the means ± SDs. Different letters indicate significant differences among the treatments at *p* < 0.05.

To explore the influences of different light wavelengths on the gene expression levels of key enzymes involved in the anthocyanins biosynthesis pathway, the quantifications of mRNA in fruits at 60 d were determined through qRT-PCR analysis. *VcC4H*, *Vc4CL*, *VcCHI*, *VcLDOX*, *VcDFR* and *VcUFGT* are vital genes in regulating anthocyanin biosynthesis of blueberry. Interestingly, the expression levels of most of these genes in the B treatment were significantly higher in blueberry fruits compared with other treatments (Figure 4). The expression level of *VcC4H* in the B treatment was significantly higher relative to other treatments, and those of the other three treatments significantly declined (Figure 4A). Similarly, the *Vc4CL* expression level significantly increased under the B treatment and was highly inhibited by other light wavelengths in fruits (Figure 4B). In addition, the expression of *VcCHI* was significantly upregulated by B light in blueberry fruits, but the minimum expression level was observed in the W treatment (Figure 4C). However, the maximum expression level of *VcLDOX* was found in the Y treatment, followed by the R treatment, and those of the W and B treatments were rapidly downregulated (Figure 4D). Moreover, the expression of *VcDFR* was significantly induced by B light in fruits, whereas those of the other three treatments were significantly inhibited (Figure 4E). Similarly, the maximum expression level of *VcUFGT* was seen in the B treatment, followed by the W and Y treatments, and that of the R treatment displayed the minimum expression level (Figure 4F).

### 3.6. Correlation and Principal Component Analysis (PCA)

**Figure 5:**
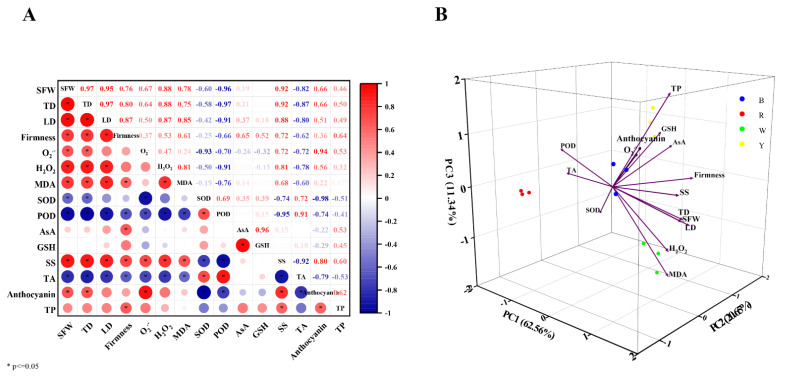
(**A**) Correlation matrix of different appearance and physiological parameters in blueberry fruit at 60 d. SFW, fruit weight; TD, fruit width; LD, fruit height; firmness; O_2_^·−^, superoxide anion radical; H_2_O_2_, hydrogen peroxide; MDA, malondialdehyde; SOD, superoxide dismutase; POD, peroxidase; AsA, ascorbic acid; GSH, glutathione; SS, soluble solids; TA, total acid; anthocyanin; TP, total phenol. The color intensity and circle size are proportional to the value of each correlation coefficient. Red represents a positive correlation, and blue represents a negative correlation. * represents a significant correlation at the 0.05 level. (**B**) PCA score plots of different appearance and physiological parameters in blueberry fruit at 60 d.

To better understand the correlations between different appearance and physiological parameters in blueberry fruit, a correlation analysis was carried out by calculating the Pearson correlation coefficient to analyze linear correlations. Under different light wavelengths, the correlations between different parameters significantly varied (Figure 5A). In general, most of these appearance and physiological parameters were positively correlated with each other in fruits. However, total acid content, SOD and POD activities were negatively correlated with most parameters, and they were significantly negatively correlated with anthocyanin content in blueberry fruits. Conversely, there were significantly positive correlations among the total acid content, SOD and POD activities. Moreover, anthocyanin content was significantly positively correlated with total phenol content, soluble solids content and some fruit appearance indexes. Interestingly, total phenol content had a positive correlation with AsA and GSH contents in fruits, whereas anthocyanin content was negatively correlated with the two parameters. 

The PCA was performed on the 15 parameters of blueberry fruit under different light-wavelength treatments, including appearance indexes, flavors indexes, antioxidant capacity, antioxidant enzyme activity and bioactive substances levels. Three principal components (PC1, PC2 and PC3) were observed when eigenvalues were greater than 1. The sum of their cumulative variance contribution was 94.55%, and 62.56%, 20.65% and 11.34% of variance was explained by PC1, PC2 and PC3, respectively (Table 3). This result showed that the information of these parameters could be effectively covered by the three principal components. Moreover, scatter points contained in each treatment were significantly separated, indicating that different light wavelengths could significantly influence these parameters in blueberry fruits (Figure 5B). Total acid content, SOD and POD activities were significantly negatively correlated with PC1, but fruit appearance indexes (fruit weight, fruit width, fruit height and firmness) and anthocyanin content had a significantly positive correlation with PC1. In addition, AsA and GSH contents were significantly positively correlated with PC2, but there were no significant correlations between other parameters and PC2. Anthocyanin and total phenol contents had a significantly positive correlation with PC3, while MDA content was significantly negatively correlated with PC3. Therefore, the results of correlation analysis and PCA both indicated that total acid content, AsA and GSH contents, SOD and POD activities, anthocyanin and total phenol contents were closely related to the blueberry fruit quality response to different light wavelengths.

## 4. Discussion

Flavor, fruit appearance and size are important commercial characteristics of blueberry fruits [1]. Fruit size is a vital factor for evaluating fruit quality, and larger fruit tends to mean higher fruit quality. Moreover, the most obvious influence of light on fruit growth and development is usually reflected in the appearance of fruits [9,13]. In this experiment, the B and W treatments had the maximum values of fruit width, fruit height and fruit weight in blueberry fruits, whereas the minimum values of the three indexes were all observed in the R treatment. These results showed that blueberry plants could produce larger fruits when they were exposed to blue light or white light, but they could produce smaller fruits when red light was used as the light source. These differences may be caused by the effects of light wavelength on photosynthesis and carbon assimilation and allocation in blueberry [31]. However, there were no changes in the value of fruit shape index among the treatments, suggesting that light wavelength may have little effect on blueberry fruit shape. In addition, light wavelength also had a significant effect on the firmness of blueberry fruits. We found that the minimum value of firmness was observed in the fruits treated with red light, which was significantly lower compared to other treatments. This result of firmness revealed that red light may lead to lower firmness of blueberry fruits, which is not conducive to the preservation of ripe fruits. Furthermore, it has been reported that there were changes in soluble solids and acid contents of fruits under different light-wavelength treatments [32,33,34]. Generally, sweetness has a positive correlation with soluble solids content in fruits, and sourness has a positive correlation with total acidity [35]. The unique flavor of blueberry fruits is one of its features that attract consumers, and it largely depends on the soluble solids and acid contents as well as their ratio in fruits [36]. Our results revealed that the R treatment had the maximum content of total acid and the minimum content of soluble solids, indicating that a significantly lower value of solid–acid ratio was obtained in the R treatment relative to the other treatments, whereas the fruits treated with blue light displayed the opposite trend. Consequently, a sweeter taste was found in blueberry fruits treated with blue light, while the fruits tasted sourer when red light was used as the light source. Similarly, research in tomato and strawberry has also found that blue light can improve soluble solids content and reduce acid content in fruits [37,38,39]. However, some studies also reported that the content of soluble solids in fruits could be improved using red light or a mixture of blue and red light [33]. As the key flavor index of fruits, soluble solids and acid contents in fruits are closely related to the photosynthesis of plants. It has been demonstrated that different light wavelengths exhibit different impacts on the photosynthesis of plants. For example, blue light has a positive influence on chloroplast development, chlorophyll formation, stomata opening and leaf expansion to strengthen the photosynthetic rate of plants [40], and red light can also promote photosynthesis in grape skins and thus improve the level of soluble sugar in fruits [13]. Previously, researchers have reported that blueberry photosynthesis is sensitive to environment conditions, and the response of blueberry fruits to different light wavelengths have varied [2,41,42]. Therefore, we speculated that compared to other light wavelengths, blue light can better promote blueberry photosynthesis to produce carbohydrates in plants and accelerate the output of photosynthetic products from leaves to fruits [43], resulting in larger fruit and higher soluble solids content in fruits. Interestingly, white light and yellow light also displayed different impacts on the contents of soluble solids and total acid in fruits, suggesting that blueberry fruits with more distinctive flavor can be produced by changing light wavelength.

When plants are under stress, the generation of reactive oxygen species (ROS) is accelerated, thus inducing oxidative stress and leading to cellular damage, while plants can scavenge ROS and reduce oxidative stress through the antioxidant defense system mainly composed of antioxidant enzymes and nonenzymatic antioxidants to ensure the normal physiological metabolic activities of plants [44,45]. As is well-known, different light wavelengths play different roles in the antioxidant capacities of fruits. Excessive accumulation of ROS such as O_2_^·−^ and H_2_O_2_ in plant cells leads to lipid peroxidation and produces MDA and other toxic products, which further damages the structure and stability of cell membranes [46]. This study has shown that the R and Y treatments maintain the lowest levels of O_2_^·−^, H_2_O_2_ and MDA during the whole fruit development period and attain levels significantly lower in comparison to other treatments, indicating that red light and yellow light can reduce the accumulation of O_2_^·−^, H_2_O_2_ and MDA in blueberry fruit cells, which is beneficial to the integrity of cell membranes. Similarly, it was also revealed that red light can significantly reduce the contents of H_2_O_2_ and MDA in tomato fruit relative to blue light and white light at different developmental stages [44]. SOD and POD are vital antioxidant plant enzymes that can play important roles in ROS scavenging and oxidative stress [45]. Our results displayed that the maximum activities of SOD and POD in blueberry fruits were both seen in the R treatment, and significantly lower SOD and POD activities were observed in the B treatment relative to other treatments. However, some studies reported that blue light could better stimulate the antioxidant system and have a more positive influence on antioxidant enzyme activities in fruits [45,47]. AsA and GSH are important nonenzymatic antioxidants, which contribute to the strength of the antioxidant systems in fruits [46]. Zhang et al. found that blue light induced the expression of AsA and GSH biosynthesis genes, thus improving the levels of AsA and GSH in citrus fruits [48]. The AsA content also increased in Chinese kale and pak-choi baby leaves under blue-light irradiation [49]. Interestingly, we found that blueberry fruits under the Y treatment had significantly higher AsA and GSH contents compared to other treatments in this experiment, suggesting that blueberry fruit can produce more AsA and GSH when yellow light is used as the light source. In summary, these results revealed that red light and yellow light can better improve the antioxidant capacity of blueberry fruit to reduce the accumulation of O_2_^·−^, H_2_O_2_ and MDA and lower oxidative stress in fruit, thus better promoting the growth and development of fruit, which may be due to red light and yellow light acting as signals to active antioxidant systems and stimulate the expression of genes related to antioxidant enzymes and nonenzymatic antioxidants biosynthesis in blueberry fruits [47]; however, their specific regulatory mechanisms may be different. Red light may induce the expression of SOD and POD biosynthesis genes in blueberry fruits, thus improving the activities of SOD and POD, and yellow light could also upregulate the expression of AsA and GSH biosynthesis genes to increase the contents of AsA and GSH in fruits, which contributed to the antioxidant capacity of blueberry fruits. Conversely, white light and blue light may fail to effectively stimulate the expression of genes related to antioxidant enzymes and nonenzymatic antioxidants biosynthesis in fruits, resulting in the accumulation of O_2_^·−^, H_2_O_2_ and MDA in blueberry fruits.

Polyphenol compounds are key nutrients and bioactive substances in blueberry fruits, which play vital roles in the antioxidant system [2]. Previous researchers have reported that light wavelength had a significant impact on the level of polyphenol compounds in fruits, especially blue light, which could effectively induce the biosynthesis of polyphenols [50]. In this experiment, we observed that significantly higher total phenol content was obtained in the Y and B treatments relative to other treatments, indicating that yellow light and blue light can better increase total phenol levels in blueberry fruits compared to white light and red light. This is in accordance with the research on strawberries, which indicated that the total phenol content was significantly improved when blue light was used as the light source [45]. He et al. also demonstrated that blue light significantly enhanced total phenol levels in tomato fruits [47]. However, there is little research on the influence of yellow light on polyphenol compounds in fruits, and its regulatory mechanism is still unclear, thus warranting further study [6,13,15]. Anthocyanins are the main component of polyphenol compounds and colored pigments in fruits, which exhibit a variety of health benefits, such as antioxidant, anticancer and other preventive properties [2,6]. Blueberry fruits are rich in anthocyanins and have attractive colors, which are welcomed and concerned by consumers. Generally, light can induce the production of anthocyanin in fruits, and light wavelength has a strong impact on the biosynthesis of anthocyanin [18,51,52]. Blue light and red light not only enable plants to produce optimal chlorophyll uptake and photosynthetic efficiency, but also seem to promote anthocyanin biosynthesis in horticultural crops more significantly [19,53,54]. For instance, Kokalj et al. found that blue light could significantly improve anthocyanin content in sweet cherries [50]. Likewise, red light has also been demonstrated to increase the level of anthocyanin in cranberry fruits compared to white light [55]. In our study, the B treatment had the maximum content of anthocyanin in blueberry fruits at levels significantly higher than that of other treatments, followed by W and Y treatments; however, significantly lower anthocyanin content was seen in the R treatment compared to other treatments. These results revealed that compared to white light, blue light displayed a more positive impact on the anthocyanin accumulation in blueberry fruits. The effect of yellow light on anthocyanin levels in fruits is not obvious, but red light may inhibit the biosynthesis of anthocyanin in fruits. This is consistent with the research on pear, strawberry and grape, which suggested that blue light contributed to the biosynthesis and accumulation of anthocyanin in fruits [5,56,57]. Interestingly, it has been reported that SOD, POD and other antioxidant substances could degrade anthocyanins, thus reducing the accumulation of anthocyanins in plants [58,59]. Here, we speculated that blueberry fruits treated with red light maintained relatively higher antioxidant enzyme activity during the whole fruit development period, thus degrading part of the anthocyanins, resulting in a reduction of anthocyanin content in fruits. 

Moreover, light wavelength also significantly regulates the expression pattern of anthocyanin biosynthesis genes in plants. Some research has revealed that blue light and red light both improve the expression levels of genes in the anthocyanin biosynthesis pathway in fruits, and red light may promote anthocyanin biosynthesis in plants better [54]. For instance, one study on bilberry exhibited that the expression levels of genes related to anthocyanin biosynthesis could be upregulated using red light and blue light in ripe fruits [6]. In particular, the expression level of UFGT was significantly improved using red light, which plays a crucial role in the last step of anthocyanin biosynthesis. Conversely, blue light could enhance the expression level of CHI, DFR, C4H, 4CL and other anthocyanin biosynthesis genes in most plants [47,60,61,62]. In this study, we found that most anthocyanin biosynthesis gene expression levels were significantly upregulated in the B treatment relative to other treatments, including *VcC4H*, *Vc4CL*, *VcCHI*, *VcDFR* and *VcUFGT*, whereas the maximum expression level of *VcLDOX* was seen in the Y treatment, indicating that blue light may better induce the expression of anthocyanin biosynthesis genes, thus resulting in the accumulation of anthocyanin in blueberry fruits. Similarly, Zhang et al. also demonstrated that blue light could promote the expression of anthocyanin biosynthesis genes in blueberry, thus improving anthocyanin content in leaves [8]. This is consistent with the result that the maximum content of anthocyanin was observed in fruits treated with blue light in this study, which again proved that blue light can better improve the level of anthocyanin in blueberry fruits. Therefore, an analysis of the expression levels of anthocyanin biosynthesis genes under different light wavelengths can also explain why the highest anthocyanin content was seen in blueberry fruits treated with blue light. In addition, the maximum expression of *VcLDOX* under the Y treatment may be the reason for the maximum content of total phenol seen in blueberry fruits treated with yellow light. As crucial secondary metabolites, polyphenols and anthocyanins are also deeply affected by plant photosynthesis. Some studies have reported that compared to other light wavelengths, photosynthetic pigments are more likely to absorb blue light, thus promoting the synthesis of lycopene in tomatoes [47,63]. Therefore, one possible explanation for the results of this experiment is that, compared to other light wavelengths, the peels of blueberry fruits are sensitive to blue light and can absorb blue light more effectively, thus enhancing the photosynthesis of fruits and improving the levels of anthocyanin in blueberry fruits. Light can also be transduced as a signal to downstream factors via photoreceptors, thus affecting the process of fruit coloring, the expression of genes and the metabolism of secondary metabolites [64]. Ni et al. revealed that blue light can promote the expression of anthocyanin biosynthesis genes through its signaling pathway, thus increasing anthocyanin levels in mango peels [9]. It has also been demonstrated that the expression level of blue light photoreceptors has a positive correlation with the level of anthocyanin [5]. Furthermore, blue light could cause the overexpression of CRYs and PHOTs to improve the content of anthocyanin in plants [65]. Therefore, another possible explanation is that blue light may be transduced as a signal via CRYs and PHOTs to induce the expression of anthocyanin biosynthesis genes, and blue light could also stimulate the expression of CRYs and PHOTs, thus resulting in the accumulation of anthocyanin in blueberry fruits. However, the specific mechanism and signaling pathway of how blue light regulates anthocyanins biosynthesis in blueberry fruits still need further research.

**Figure 6:**
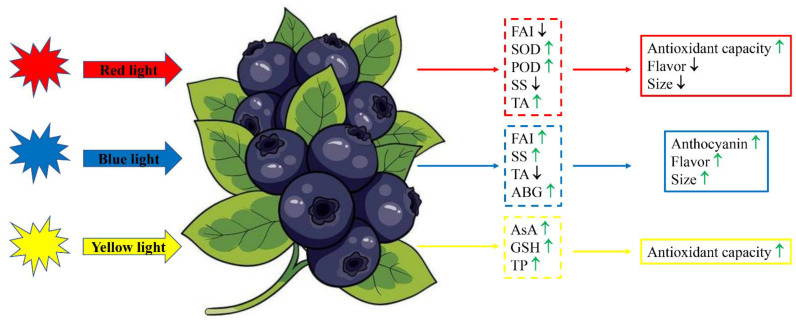
Possible mechanism of regulating fruit quality and anthocyanin content in blueberry via different light wavelengths. The green up-arrow indicates a significant increase, and the black down-arrow indicates a significant decrease. FAI, fruit appearance indexes; SOD, superoxide dismutase; POD, peroxidase; SS, soluble solids; TA, total acid; ABG, anthocyanin biosynthesis genes; AsA, ascorbic acid; GSH, glutathione; TP, total phenol.

The regulatory mechanism of light wavelength on fruit quality, growth and development is very complex, and neither individual nor multiple indicators can effectively clarify the physiological and metabolic activities involved in this process. Therefore, correlation analysis and PCA were performed on blueberry fruit appearance and physiological parameters in this study. These results displayed that total acid content, SOD and POD activities were significantly negatively correlated with anthocyanin content, but total phenol and soluble solids contents were significantly positively correlated with anthocyanin content in blueberry fruit. Three principal components were extracted from 15 blueberry fruit quality and growth parameters, and PC1 was significantly correlated with total acid content, SOD and POD activities, anthocyanin content and fruit appearance indexes, indicating that these indicators were closely related to the blueberry fruit quality under different light wavelengths. In a word, the impacts of different light wavelengths on blueberry fruit quality and anthocyanin content were significantly different; in particular, blue light could better improve the appearance and anthocyanin content of blueberry fruit, whereas red light and yellow light had a more positive influence on antioxidant capacity in fruit (Figure 6).

## 5. Conclusions

In this study, we explored the influence of different light wavelengths on blueberry fruit quality and anthocyanin content. Our results showed that the B and W treatments attained the maximum values of fruit width, fruit height and fruit weight in blueberry fruits, whereas the R treatment showed the opposite trends. Moreover, the R treatment attained the maximum activities of SOD and POD, and the Y treatment displayed the maximum contents of AsA, GSH and total phenol in fruits, thus improving blueberry fruit antioxidant capacity. Interestingly, total acid content, SOD and POD activities were significantly negatively correlated with anthocyanin content in fruits. The solidity–acid ratio of blueberry fruit differed under different light wavelengths, and the sweetest flavor was obtained in the B treatment, while the R treatment had the sourest flavor in fruits. Finally, blue light could significantly upregulate the expression levels of anthocyanin biosynthesis genes and increase the content of anthocyanin in blueberry fruits, thus resulting in the accumulation of anthocyanin in fruits. Therefore, blue light irradiation may be an effective way to increase anthocyanin content of blueberry fruit in practice. These results provide new insights for the regulation of blueberry fruit quality formation and anthocyanin biosynthesis via different light wavelengths.

## Figures and Tables

**Table 1:** Sequences of all primers used in the qRT-PCR.

Gene Name	Gene ID	Primer Sequence (5′ to 3′)
VcC4H	VaccDscaff24-augustus-gene-58.38	F: TTGGTGTCGGTAGGAGGAGTTG
		R: TCAGAACGATGGTAGAGTGCTTCA
Vc4CL	VaccDscaff34-processed-gene-57.9	F: TTGTGAGGAACGCCGAGATGAA
		R: AATGTAGCCTATGTCGCCAGTGT
VcCHI	VaccDscaff29-processed-gene-189.2	F: GGCACCACCAATTCCTTCTTCC
		R: TAACGGCGAGCGACTCTACG
VcLDOX	VaccDscaff43-augustus-gene-236.29	F: CCGTGGAGGAGAAGGAGAAGT
		R: GGTGATTCGCATACTCGCTTGT
VcDFR	VaccDscaff1613-processed-gene-0.0	F: CATTCAAGGTTGTGCTGGTGTCT
		R: ACGGTTCCTGTGGTTGAAGTGTA
VcUFGT	VaccDscaff1-augustus-gene-318.39	F: GCTGGAATCCTATGCGGTCAAG
		R: ATCTCCTCCTCCTCCTCCTCATT

**Table 2:** Effects of different light wavelengths on fruit appearance indexes.

Parameter	Treatment	Days after Treatment/(d)				
		0	15	30	45	60
Fruit weight/(g)	W	0.16 ± 0.03 a	0.60 ± 0.08 b	0.61 ± 0.10 bc	0.86 ± 0.11 b	2.28 ± 0.25 a
	R	0.14 ± 0.03 a	0.63 ± 0.09 ab	0.56 ± 0.06 c	0.64 ± 0.08 c	1.21 ± 0.46 c
	B	0.15 ± 0.02 a	0.57 ± 0.09 b	0.66 ± 0.08 b	0.80 ± 0.06 b	2.31 ± 0.32 a
	Y	0.16 ± 0.05 a	0.68 ± 0.11 a	0.75 ± 0.14 a	1.04 ± 0.15 a	1.77 ± 0.17 b
Fruit width/(mm)	W	6.78 ± 0.53 a	10.18 ± 0.50 b	10.84 ± 0.64 bc	12.35 ± 0.59 b	16.79 ± 0.76 a
	R	6.45 ± 0.42 a	10.06 ± 0.88 b	10.73 ± 0.41 c	11.09 ± 0.47 c	13.32 ± 1.31 c
	B	6.64 ± 0.50 a	10.54 ± 0.81 ab	11.31 ± 0.53 ab	11.97 ± 0.43 b	16.90 ± 0.93 a
	Y	6.64 ± 0.44 a	10.92 ± 0.64 a	11.83 ± 0.69 a	13.23 ± 0.75 a	15.18 ± 0.55 b
Fruit height/(mm)	W	5.58 ± 0.58 a	8.32 ± 0.75 a	8.62 ± 0.52 ab	9.02 ± 0.65 b	13.72 ± 0.60 a
	R	5.09 ± 0.40 a	7.52 ± 0.61 b	7.89 ± 0.38 c	8.50 ± 0.28 c	10.48 ± 1.56 c
	B	5.28 ± 0.47 a	8.30 ± 0.71 a	8.40 ± 0.44 b	8.86 ± 0.48 bc	13.23 ± 0.76 ab
	Y	5.28 ± 0.61 a	8.71 ± 0.77 a	9.00 ± 0.47 a	9.67 ± 0.71 a	12.41 ± 0.61 b
Fruit shape index	W	0.82 ± 0.07 a	0.82 ± 0.04 a	0.80 ± 0.05 a	0.73 ± 0.06 b	0.82 ± 0.02 a
	R	0.79 ± 0.04 a	0.75 ± 0.03 b	0.74 ± 0.04 b	0.77 ± 0.03 a	0.78 ± 0.06 a
	B	0.80 ± 0.09 a	0.79 ± 0.03 ab	0.74 ± 0.05 b	0.74 ± 0.04 ab	0.78 ± 0.04 a
	Y	0.80 ± 0.07 a	0.80 ± 0.05 ab	0.76 ± 0.04 ab	0.73 ± 0.03 b	0.82 ± 0.04 a

Note: All values are expressed as the means ± SDs. Different letters indicate significant differences among the treatments at *p* < 0.05.

**Table 3:** Eigenvalues of each principal component.

Trait	Component		
	1	2	3
SFW	1.50 **	0.13	−0.40
TD	1.51 **	0.15	−0.37
LD	1.44 **	0.43	−0.58
Firmness	1.18 **	0.83	0.18
O_2_^·−^	1.18 **	−0.65	0.95
H_2_O_2_	1.34 **	−0.02	−0.96
MDA	1.10 **	0.28	−1.62 **
SOD	−1.13 **	0.79	−0.98
POD	−1.52 **	0.14	0.47
AsA	0.22	1.38 **	0.57
GSH	−0.03	1.33 **	0.81
SS	1.52 **	0.00	0.12
TA	−1.44 **	0.21	−0.03
Anthocyanin	1.24 **	−0.63	1.07 **
TP	0.93	0.44	1.81 **
Total	9.05	4.78	1.04
% of variance	62.56	20.65	11.34
Cumulative %	62.56	83.21	94.55

Note: ** represents eigenvalues are significant ≥ 1.00.

## Data Availability

The data presented in this study are available on request from the corresponding authors. The data are not public.
